# Supplementary material for: Global folate deficiency among adolescent girls: A systematic review and meta-analysis
Source: PLoS One. 2026 Apr 20;21(4):e0346599. doi: 10.1371/journal.pone.0346599 (PMC13094969; doi:10.1371/journal.pone.0346599)
Supplement: S1 File — (DOCX) [file pone.0346599.s001.docx]

**Supplementary 3. Folate Deficiency and Its Associated Factors Among Adolescent Girls: A Systematic Review and Meta-analysis**

| ***Type of database*** | ***Number of articles identified*** | | ***Search Terms For Folate Deficiency and Its Associated Factors Among Adolescent Girls: A Systematic Review and Meta-analysis*** | ***Accessed Date and time*** |
| --- | --- | --- | --- | --- |
|  |  | |  |  |
| ***Pub med*** | ***1021*** | ***1*** | ***("Folic Acid Deficiency"[MeSH] OR "folate deficiency"[Title/Abstract] OR "folic acid deficiency"[Title/Abstract] OR "low folate"[Title/Abstract] OR "serum folate"[Title/Abstract] OR "RBC folate"[Title/Abstract] OR "erythrocyte folate"[Title/Abstract] OR "folate insufficiency"[Title/Abstract] OR "folate status"[Title/Abstract] OR "micronutrient deficiency"[Title/Abstract] OR "nutrient deficiency"[Title/Abstract] OR "vitamin deficiency"[Title/Abstract])*** | ***2025-09-02, 11:45 EAT*** |
|  |  | ***2*** | ***(adolescent[Title/Abstract] OR adolescents[Title/Abstract] OR teenager[Title/Abstract] OR teenagers[Title/Abstract] OR youth[Title/Abstract] OR girl[Title/Abstract] OR girls[Title/Abstract] OR female[Title/Abstract] OR "adolescent girls"[Title/Abstract] OR "female adolescents"[Title/Abstract])*** |  |
|  |  | ***3*** | ***#1 AND #2*** |  |
| ***Hinari*** | ***47*** | | *TitleCombined:( "folic acid deficiency" OR "folate deficiency" OR "low folate" OR "serum folate" OR "RBC folate" OR "erythrocyte folate" OR "folate insufficiency" OR "folate status")*  *AND*  *TitleCombined:( adolescent* OR teenager* OR youth OR girl* OR female* OR "adolescent girls" OR "female adolescents")* | 2025-09-03, 9:35 EAT |
| ***Science direct*** | ***268*** | | *("folic acid deficiency" OR "folate deficiency" OR "low folate" ) AND ("adolescent Girls" OR "female adolescents" OR "teenager Girls" )* | 2025-09-03, 10:04 EAT |
| ***DOAJ*** | ***127*** | | *("folate deficiency" OR "folic acid deficiency" OR "low folate" OR "serum folate" OR "RBC folate" OR "erythrocyte folate" OR "folate insufficiency" OR "folate status")*  *AND*  *(adolescent OR adolescents OR teenager OR teenagers OR youth OR girl OR girls OR female OR "adolescent girls" OR "female adolescents")* | ***2025-09-02, 11:45 EAT*** |
| ***Google and google scholar*** | ***35*** | | ***"folate deficiency" OR "folic acid deficiency" OR "low folate" AND adolescent OR teenager OR girl OR "female adolescent"*** | ***2025-09-02, 11:45 EAT*** |
| ***Total*** | ***1498*** | |  |  |
