# Supplementary material for: Global folate deficiency among adolescent girls: A systematic review and meta-analysis
Source: PLoS One. 2026 Apr 20;21(4):e0346599. doi: 10.1371/journal.pone.0346599 (PMC13094969; doi:10.1371/journal.pone.0346599)
Supplement: S3 File — (DOCX) [file pone.0346599.s003.docx]

Supplementary 3. Methodological quality assessment of included studies using Newcastle-Ottawa Scale (NOS)

| **No.** | **Author** | **Representativeness** | **Sample size** | **Non-respondents** | **Ascertainment of the exposure** | **comparability** | **Assessment of outcome** | **Statistical test** | **Quality score** |
| --- | --- | --- | --- | --- | --- | --- | --- | --- | --- |
| 1 | Abdelrahim II et al. | 0 | 0 | 0 | 1 | 1 | 2 | 1 | 5 |
| 2 | Ahmed F et al. | 0 | 0 | 0 | 1 | 1 | 2 | 1 | 5 |
| 3 | Awasthi S et al. | 1 | 1 | 0 | 1 | 1 | 2 | 1 | 7 |
| 4 | Bansal PG et al. | 1 | 1 | 0 | 1 | 0 | 2 | 1 | 6 |
| 5 | Basiry M et al. | 1 | 0 | 0 | 1 | 0 | 2 | 1 | 5 |
| 6 | Daniel Jr WA et al. | 1 | 0 | 0 | 1 | 1 | 2 | 1 | 6 |
| 7 | de Lanerolle-Dias et al. | 1 | 1 | 1 | 1 | 1 | 2 | 1 | 8 |
| 8 | Demuyakor ME et al. | 1 | 1 | 0 | 1 | 0 | 2 | 1 | 6 |
| 9 | Dhurde VS et al. |  | 0 | 0 | 1 | 1 | 2 | 1 | 6 |
| 10 | Ercan S et al. | 1 | 0 | 1 | 1 | 1 | 2 | 1 | 7 |
| 11 | Ethiopian Public Health Institute (EPHI) | 1 | 1 | 0 | 1 | 0 | 2 | 1 | 6 |
| 12 | Htet MK et al. | 1 | 0 | 1 | 1 | 0 | 2 | 1 | 6 |
| 13 | Jani R. et al. | 1 | 0 | 0 | 1 | 1 | 2 | 1 | 6 |
| 14 | Karakaş NM et al. | 0 | 1 | 1 | 1 | 1 | 2 | 1 | 7 |
| 15 | Kumar KJ et al. | 1 | 0 | 1 | 1 | 0 | 2 | 1 | 6 |
| 16 | Liebman M | 1 | 0 | 1 | 1 | 0 | 2 | 1 | 6 |
| 17 | Monge-Rojas et al. | 1 | 0 | 1 | 1 | 0 | 2 | 1 | 6 |
| 18 | Öner et al. | 1 | 1 | 1 | 1 | 1 | 2 | 1 | 8 |
| 19 | Reiter LA et al. | 1 | 0 | 0 | 1 | 0 | 2 | 1 | 5 |
| 20 | Saxena R et al. | 1 | 1 | 0 | 1 | 1 | 2 | 1 | 7 |
| 21 | Shalini T et al. | 1 | 1 | 1 | 1 | 0 | 2 | 1 | 7 |
| 22 | Thoradeniya T et al. | 1 | 0 | 1 | 1 | 1 | 2 | 1 | 7 |
| 23 | Tsui JC et al. | 0 | 0 | 1 | 1 | 0 | 2 | 1 | 5 |
| 24 | VanderJagt DJ et al. | 0 | 0 | 1 | 1 | 1 | 2 | 1 | 6 |
| 25 | Clark et al. | 0 | 0 | 1 | 1 | 1 | 2 | 1 | 6 |
| 26 | Doshi et al. | 0 | 1 | 1 | 1 | 1 | 2 | 1 | 7 |
