# Supplementary material for: Global folate deficiency among adolescent girls: A systematic review and meta-analysis
Source: PLoS One. 2026 Apr 20;21(4):e0346599. doi: 10.1371/journal.pone.0346599 (PMC13094969; doi:10.1371/journal.pone.0346599)
Supplement: Attachment — Submitted filename: Resposnse to reviewer.docx [file pone.0346599.s006.docx]

**29 Jan, 2026**

**Dear Editors of PLOS ONE,**

We thank you and the reviewers for the time, effort, and expertise dedicated to the evaluation of our manuscript, PONE-D-25-60905 , entitled “Global Folate Deficiency Among Adolescent Girls: A Systematic Review and Meta-analysis.” We greatly appreciate the constructive and insightful comments provided, which have been invaluable in improving the clarity, rigor, and overall quality of the manuscript.

In response to the reviewers’ feedback, we have carefully revised the manuscript and prepared a detailed, point-by-point response explaining how each comment has been addressed. All modifications have been clearly indicated in the revised manuscript to facilitate review. We believe that these revisions have strengthened the analysis, interpretation, and presentation of our findings.

Thank you for the opportunity to revise and resubmit our work. We appreciate your consideration and look forward to your further assessment.

**Sincerely**

**Mekuriaw Nibret Aweke**

**Corresponding Author**

1. **Editors comment**

The overall topic is of value in Public Health Nutrition. However, there are many important methodological aspects for a major review as pointed out by the reviewers. A special attention should be provided for the following recommendations: • The inclusion of Google and Google Scholar as primary data sources raises concerns about reproducibility; • It is not clear whether controlled vocabulary terms (e.g., MeSH terms) were used in PubMed or whether the search relied exclusively on free-text terms; and • The Methods section would benefit from explicitly stating the review question (e.g., using a PECO or similar framework). Please submit your revised manuscript

Authers response:

Thank you for the opportunity to revise our manuscript in response to the editors’ and reviewers’ comments and suggestions. We have carefully revised the manuscript, with particular attention to strengthening the search strategy through refinement of the search terms (including MeSH terms) and by explicitly stating the review question using the PICO framework. We believe these revisions have improved the methodological clarity and robustness of the manuscript.

1. Please ensure that your manuscript meets PLOS ONE's style requirements, including those for file naming.

Authers response:

Dear editor, thank you very much for the reminder to meets the PLOS ONE’S style requirement. We have revised the manuscript in accordance with the journals style requirement including file naming.

2. We note you have included a table to which you do not refer in the text of your manuscript. Please ensure that you refer to Table 4 in your text; if accepted, production will need this reference to link the reader to the table.

Authers response:

Dear editor, thank you very much for your request to refer table 4 in the text. We noted that we didn’t refer table 4 and now we have revised it and we ensure that all the table in the manuscript were correctly referred. Thank you for your request.

3. Please include captions for your Supporting Information files at the end of your manuscript, and update any in-text citations to match accordingly.

Authers response:

Dear revewer, thank you very much you’re your request to add supporting information files at the end of our manuscript. We have added the supporting information files at the end of our manuscript.

1. **Revewer #1 comments**

Relevant and well-written article. Just a few points to be reviewed:

**Response to Reviewer:**

Thank you for your positive feedback on the relevance and importance of our study. We value your comments and suggestions, which have helped us improve the manuscript.

1. Abstract

The results do not indicate how many studies were captured in the search strategy and how many were included, which is necessary according to the PRISMA-2020 checklist guidelines for abstracts.

-In conclusions, the excerpt "Folate deficiency among adolescent girls is highly prevalent globally, affecting more than one in four adolescent girls. The burden is particularly higher in Africa, where over one-third of adolescent girls are affected." repeats exactly what was said in the results. I recommend removing it.

**Response to Reviewer:**

Dear Reviewer, Thank you for this comment. We have revised the abstract to explicitly report the number of records identified and the number of studies included, in accordance with the PRISMA-2020 checklist for abstracts. The revise statement reads as the following:

“*The search strategy identified 1,498 records, of which 26 studies met the eligibility criteria and were included in this systematic review and meta-analsyis.*”

In addition in the conclusion section we had removed the repeated sttatment which will enhance the readability of the abstract sections. Thank you very much for this crucial and insightful suggesions.

2. Methods

-The definition of adolescence can vary culturally. Indicate which age range was considered to define this life stage.

**Response to Reviewer:**

Dear Reviewer,

*Thank you for this comment. We have clarified the definition of adolescence in the Methods section. Adolescence was defined as ages 10–19 years, consistent with the World Health Organization classification, and this age range was applied as an inclusion criterion for the review.*

-In Supplementary File 1 (S1), the only table inserted is titled S3.

**Response to Reviewer:**

Dear Reviewer,

Thank you for pointing this inconsistent labeling of supplmentary files. The supplementary files were correctly labeled as Supplementary Files 1–4. We have corrected the table labeling so that each supplementary file now contains a consistently numbered table corresponding to its file number, and all in-text references have been updated accordingly.

-The inclusion and exclusion criteria lack the use of an acronym (such as PECOT) in the form of a table to more clearly indicate all the criteria.

**Response to Reviewer:**

Dear Reviewer,

Thank you for suggesting the inclusion of the PECOT framework for our eligibility criteria. We have now added a table presenting the PECOT criteria used in our study. We hope this provides a clear and concise overview of the inclusion and exclusion criteria, and we greatly appreciate your insightful recommendation.

-To assess the degree of agreement between reviewers, it would be useful to calculate Cohen's Kappa index.

**Response to Reviewer:**

Dear Reviewer, we sincerely appreciate your suggestion regarding the calculation of Cohen’s Kappa. While we did not calculate the statistic, all discrepancies between reviewers during study selection were carefully discussed and resolved by consensus, ensuring accurate and consistent inclusion and exclusion decisions.

- In the assessment of the quality of the individual studies, it is stated that a modified version of the Newcastle-Ottawa Scale for cross-sectional studies was used. What modification? Is it validated? Was it modified to evaluate non-cross-sectional studies? Why not use other tools specifically designed for this purpose?

**Response to Reviewer:**

We thank the reviewer for this important point. We used a modified version of the Newcastle–Ottawa Scale (NOS) adapted for cross-sectional studies, following published guidance in the literature. The modifications involved adapting the selection and outcome assessment items to be applicable to cross-sectional designs, while retaining the core scoring system for representativeness, sample size, comparability, and outcome assessment.

This modified tool has been widely used and cited in systematic reviews of prevalence studies. It was not applied to non-cross-sectional studies. We selected this tool because it allows structured and reproducible assessment of methodological quality in observational studies.

-In data extraction, it would be interesting to determine if the studies have conflicts of interest.

**Response to Reviewer:**

We thank the reviewer for the suggestion to report potential conflicts of interest. We carefully reviewed each included study and did not identify any reports of conflicts of interest.

3. Results
-In Table 1:
a. The studies are ordered alphabetically. This is not incorrect, but it is generally done by the year of publication of the studies, establishing a chronology that facilitates understanding the evolution of the research.

**Response to Reviewer:**

We thank the reviewer for valuable recommendation to order the studies based on year of publication. We have revised the table and studies are ordered based on the publication year and we hope new it becomes easier to understand the evolution of the research. Thank you very much for this crucial comment.
b. There is a lack of standardization in the table, with no initial capital letter for "Mean of age" and "Type of study" in the last two included studies.

**Response to Reviewer:**

We thank the reviewer for the valuable recommendation to standardize the table by correcting the initial capital letters for "Mean of Age" and "Type of Study." We have now standardized the table by capitalizing the initial letter of each phrase or word in all table columns and rows. We hope this improves the table’s consistency and readability.
c. It is important to indicate the reference number in the first column or in parentheses next to the authors' names.

**Response to Reviewer:**

We thank the reviewer for this valuable suggestion. In response, we have updated the table to include references using the “Author (Reference)” format as recommended.

-In Table 5 of the GRADE assessment, although almost all studies were cross-sectional, separating them by design, rather than simply grouping them all as observational, enriches the reader's understanding.

**Response to Reviewer:**

We thank the reviewer for this valuable suggestion. We acknowledge that although the majority of our included studies were cross-sectional (25 out of 26), separating them by study design rather than grouping all as “observational” provides clearer information. In response, we have updated Table 5 of the GRADE assessment to present the cross-sectional studies and the single longitudinal l study separately, allowing a more precise understanding of the evidence quality for each design.

**Reviewer #2: General Comments**
The study has several strengths, including adherence to the PRISMA guidelines, prospective registration in PROSPERO, and the availability of complete search strategies for each database. These aspects contribute positively to transparency and methodological rigor. However, some methodological aspects still require clarification or stronger justification to ensure reproducibility and alignment with best practices for prevalence meta-analyses.

**Response to Reviewer:**

We appreciate the positive feedback and explanation about the methodological rigor. We have revised the document in each section including methodological clarification by including stronger justification to ensure reproducibility and alignments. We hope this will enhance the quality of the work.

Abstract
• The abstract does not provide sufficient methodological detail for readers to properly assess the robustness of the review.

**Response to Reviewer:**

We thank the reviewer for this insightful comment. In response, we have revised the abstract to include additional methodological details to enhance transparency and allow readers to better assess the robustness of the review. Specifically, the abstract methods section now states:

“Publication bias was evaluated through visual inspection of funnel plots and Egger’s regression test, and adjusted estimates were calculated using the trim-and-fill method. Meta-regression analyses were conducted to explore potential sources of heterogeneity. Sensitivity analyses were performed to assess the robustness of pooled estimates.”

• Minor grammatical and stylistic revisions are needed to improve clarity and readability.
• The objective statement contains a grammatical error (“aims to addresses”) that should be corrected.

**Response to Reviewer**

**We thank the reviewer for this valuable comment. The grammatical and typographical errors in the abstract have been corrected, and we believe that the revisions have improved the clarity and overall quality of the manuscript.**
• Percentages should be formatted consistently, with appropriate spacing and punctuation (e.g., “26.9% (95% CI: 20.5–33.2)”).

**Response to Reviewer**

**We thank the reviewer for this very valuable suggesions to use consistent writing format to report the pooled prevalence. Based on the suggesions we have correct the inconsistent punctuation and writing format throughout the manuscript and now we used** “26.9% (95% CI: 20.5–33.2) through out the document. Thank you very much for this important comment.

Introduction
• The objective of the study is not clearly stated at the end of the Introduction. The final paragraph should explicitly present the study aim to ensure coherence with the Methods and Results sections.

Response to Reviewer:

Dear reviewer thank you very much for this insightful comment to add the study objective in the introduction section. Accordingly we had added the clear and precise of this study objective at the end of the introduction section and the final sttement read as the following:

“*The objective of this study was to systematically review and quantitatively synthesize the available evidence to estimate the pooled prevalence of folate deficiency among adolescent girls and to inform targeted public health interventions.”*

Methods
The Methods section is generally well organized and follows the standard structure expected for a systematic review and meta-analysis. The authors report protocol registration, adherence to PRISMA guidelines, duplicate screening, independent data extraction, and the use of established tools for quality and certainty assessment. Overall, this reflects an adequate methodological framework. However, several points require clarification or better justification to improve transparency and reproducibility.

Response to Reviewer:

Dear reviewer, we are glad to hear this acknowledgment and consideration about the adequate methodological framework. Thank you again and we hope all other issues are adressed in the revised manusrcipt.

Many of the elements listed below appear later in the Results section, suggesting that they were considered during the review process. Nonetheless, these aspects should be clearly described in the Methods section, rather than being left implicit, to avoid ambiguity for the reader. I therefore suggest revising the Methods to improve organization and clarity.

Response to Reviewer:

Dear Reviewer, we sincerely appreciate your valuable suggestions and constructive comments regarding the Methods section. We have carefully revised the Methods section to address each of the raised concerns and clarify aspects that were previously insufficiently described. We hope that these revisions have adequately addressed the issues highlighted.
The study population is not clearly defined. The authors should explicitly state the age range used to define “adolescents” and clarify whether only healthy adolescent girls were included or whether those with underlying health conditions were also eligible.

Response to Reviewer:

Dear Reviewer, we thank you for raising these important concerns regarding clarification of the adolescent age range and the inclusion criteria related to health status. We have revised the Methods section to improve clarity by explicitly specifying that adolescent girls aged 10–19 years were included in the study and that only apparently healthy adolescent girls were considered. These details are now clearly stated in the Methods section of the revised manuscript.
• It is unclear whether the final version of the review strictly followed the registered PROSPERO protocol. The authors should clearly state whether any deviations occurred after registration (e.g., changes in eligibility criteria, outcomes, or subgroup analyses), and, if so, provide justification.

Response to Reviewer:

*We thank the reviewer for this important comment. We confirm that the final version of the review strictly adhered to the registered PROSPERO protocol. No deviations were made after registration with respect to the eligibility criteria, outcomes of interest, subgroup analyses, or planned methods. We have revised and stated clearly as follows:*

*“This review was conducted in strict accordance with the pre-registered PROSPERO protocol (Registration No:CRD420251151715) and no deviations from the protocol were made with respect to eligibility criteria, outcomes, or planned analyses.”*
• The rationale for the choice of databases should be better explained. For this topic, other widely used databases such as CINAHL, the Cochrane Library, PsycINFO, and Scopus are commonly included. In addition, the inclusion of HINARI and DOAJ alongside PubMed and ScienceDirect should be justified, given the potential overlap in indexed content.

Response to Reviewer:

Dear reviewer thank you for your inquiry to justify the data base selection. We selected PubMed and ScienceDirect for their comprehensive coverage of biomedical and health sciences literature. HINARI and DOAJ were included to ensure access to open-access research, particularly from low- and middle-income countries, reducing publication bias. While databases like CINAHL, Cochrane Library, PsycINFO, and Scopus are valuable, their content largely overlaps with PubMed and ScienceDirect, and access limitations influenced our choice.
• The inclusion of Google and Google Scholar as primary data sources raises concerns about reproducibility. Although these platforms are sometimes used to identify grey literature, the Methods section does not describe how searches were standardized (e.g., number of pages screened, sorting criteria, or limits applied).

Response to Reviewer:

Dear reviewer, we acknowledge your concern regarding the inclusion of Google and Google Scholar under the database search results. We recognize that this may have caused confusion. We have now revised the PRISMA flowchart and explicitly listed Google and Google Scholar separately under “Other Sources,” distinct from the databases. We hope this clarification makes the PRISMA flow clear.
• It is not clear whether controlled vocabulary terms (e.g., MeSH terms) were used in PubMed or whether the search relied exclusively on free-text terms. This information is important to assess the sensitivity and completeness of the search strategy.

Response to Reviewer:

We appreciate the reviewer’s comment regarding the use of controlled vocabulary. In our PubMed search, we employed a combination of both MeSH terms and free-text keywords to ensure a comprehensive and sensitive search strategy. This approach allowed us to capture relevant articles indexed under standardized terms while also including recent publications that may not yet have MeSH indexing. We have clarified this point in the revised Methods section and reads as the following:

“Papers were identified using a combination of Medical Subject Headings (MeSH), keywords, and truncations, with the following categories combined using the AND Boolean operator.”
• The Methods section would benefit from explicitly stating the review question (e.g., using a PECO or similar framework).

Response to Reviewer:

Thank you for your insightful recommendation to explicitly state the review question using a structured framework such as PECO or a similar approach. In response, we have revised the manuscript to adopt the PECOT framework and have clearly specified the research question along with the corresponding inclusion criteria and we clearly stated in the table for easliy readability. We hope that this revision adequately addresses your concern. We would be pleased to make further revisions should any additional issues remain.

• Although studies reporting folate deficiency were included, acceptable biomarkers (e.g., serum folate versus red blood cell folate) and diagnostic cut-off values are not specified. Given the large variability in cut-offs across settings and over time, this omission is important and likely contributes substantially to the extreme heterogeneity observed. While laboratory methods and cut-offs are listed as extracted variables, it remains unclear how this variability was handled analytically (e.g., subgroup analyses, sensitivity analyses, or standardization).

Response to Reviewer:

Thank you for this valuable suggestion. We conducted subgroup analyses based on the sample type(serum folate and RBC folate) and reported the results in the text and figures. Although cut-off values for serum and red blood cell folate were extracted and presented in the data extraction table, subgrouping by individual cut-off values resulted in unreadable forest plots, and few studies did not report specific cut-offs. Therefore, we performed subgroup analyses based on sample type (serum versus red blood cell folate) and reported the corresponding cut-off values in the data extraction table. We believe this approach improves the clarity and quality of the Results section. We have also acknowledged this different cut off values used in the included studies as the limitation in the manuscript. Thank you very much for your helpful suggesions.
• The inclusion of both published and unpublished studies increases comprehensiveness, but also raises concerns regarding study quality and selective availability, which should be addressed more explicitly.

Response to Reviewer:

Thank you for this valuable comment. In our inclusion criteria, we considered both published and unpublished studies. However, following the comprehensive literature search, nearly all included studies were published articles. Only one unpublished study a national report conducted by the Ethiopian Public Health Institute met the inclusion criteria. No additional unpublished studies were identified. Therefore, we believe that the potential influence of selective availability or study quality issues related to unpublished data is likely to be minimal. Thank you for your valuable comments and suggesions.
• The Methods section does not specify which version or adaptation of the Newcastle–Ottawa Scale was used, nor whether the adapted version has been validated for cross-sectional studies.

Response to Reviewer:

Thank you for this important comment. We have now clarified in the Methods section that an adapted version of the Newcastle–Ottawa Scale for cross-sectional studies was used for quality assessment. This adapted version has been widely applied in previous systematic reviews of cross-sectional studies, and we have added an appropriate reference to support its use. The revised paragraph in the methods section reads as follows:

“*Three reviewers (MNA, BAM, and TED) independently assessed the quality of the selected studies using a version of the Newcastle-Ottawa Scale modified for cross-sectional study designs. In this review, we used a version of the Newcastle–Ottawa Scale (NOS) modified for cross-sectional studies, assessing study quality across five domains: selection of study groups, sample size, non-respondents, comparability of groups, and ascertainment of the outcome (37*)…”

• The justification for using a random-effects model is generic. Given the expected heterogeneity in prevalence studies, the authors should specify which estimator was used (e.g., DerSimonian–Laird or restricted maximum likelihood [REML]).

Response to Reviewer:

Thank you very much for your valuable suggestion to specify the estimator used in the random-effects model. We have revised the Methods section to clearly indicate the estimator applied, and the forest plot now explicitly displays this information. The revised statement reads as follows:

“*Given the presence of heterogeneity among studies, a random-effects model using the DerSimonian–Laird method was applied to estimate the pooled prevalence of folate deficiency among adolescent girls.”*

• The significance level used for funnel plots and publication bias assessment should be clearly stated.

Response to Reviewer:

Dear Reviewer, we appreciate your valuable suggestion regarding the specification of the significance level for publication bias assessment. We have revised the Methods section accordingly, and the updated statement now reads as follows:

“*Publication bias was assessed using funnel plots and Egger’s regression test, with a significance level of 0.05*.”

• The authors do not report whether prevalence estimates were transformed (e.g., logit transformation) prior to pooling, which is an important methodological consideration in meta-analyses of proportions.

Response to Reviewer:

Dear reviewer thank you for the valuable comment. Prevalence estimates were pooled without transformation, as the reported results from the included studies were generally not extremely close to 0 or 100%. Given this, variance instability was minimal, and direct pooling was considered appropriate for this dataset. We refrained from applying transformations such as logit or arcsine to avoid reducing interpretability since with moderate prevalences. We are open to making corrections if any adressed important issues and we highly value the reviewer comment and suggesions.
• The use of Egger’s test to assess publication bias is questionable in this context, as it is not well suited for proportion data, particularly in the presence of extreme heterogeneity. This limitation should be acknowledged or alternative approaches considered.

Response to Reviewer:

Response to Reviewer:

We thank the reviewer for highlighting this important point. We acknowledge that Egger’s test may not be ideal for assessing publication bias in meta-analyses of proportions, particularly in the presence of substantial heterogeneity. To address this concern, we also conducted visual inspection of funnel plots and applied the trim-and-fill method, which provide complementary assessments of potential publication bias.

The revised stetement reads as the following:

“We acknowledge that Egger’s test may not be ideal for assessing publication bias in meta-analyses of proportions, particularly in the presence of substantial heterogeneity.”

Results
• The statement “Most studies were of moderate to high quality, with scores ranging from five to eight” is imprecise. The authors should report the exact number and proportion of studies in each quality category.

Response to Reviewer:

We thank the reviewer for this suggestion. Based on the quality assessment criteria, 17 studies (65%) were rated as Satisfactory (scores 5–6), 9 studies (35%) were rated as Good (scores 7–8), and no studies were rated as Very Good (9–10) or Unsatisfactory (0–4). We have updated the manuscript to include this detailed breakdown to provide a more precise and transparent description of study quality.
• Figure 1 is appropriately referenced; however, minor typographical errors should be corrected (e.g., “Defiecency,” “adolecent”).

Response to Reviewer:

Dear reviewer we appreciate your detail review and rising point by point comment to enhance the quality of this work. In response we have corrected the minor spelling issues that was found under figure 1 captions. Thank you again for your valuable comments.
• The statement that a fixed-effect model “indicated significant heterogeneity” is conceptually incorrect. Heterogeneity is assessed independently of the choice of meta-analytic model. This sentence should be revised for conceptual accuracy.

Response to Reviewer:

We thank the reviewer for pointing this out. We agree that heterogeneity should be assessed independently of the meta-analytic model. The manuscript has been revised to clarify that significant heterogeneity was observed among the included studies, and accordingly, a random-effects model was applied for all analyses.
• Given the extremely high heterogeneity, the Results section should more clearly emphasize that the pooled estimate represents an average across highly heterogeneous contexts, rather than a precise global prevalence.

Response to Reviewer:

We thank the reviewer for this important comment. We agree that the high heterogeneity among included studies limits the interpretation of the pooled estimate. The Results section has been revised to emphasize that the pooled prevalence of 26.9% (95% CI: 20.5%–33.2%) represents an average across highly heterogeneous contexts, and should not be interpreted as a precise global prevalence of folate deficiency among adolescent girls. Thank you very much for your invaluable comment and suggesions that will enhance the quality if the study.
• The interpretation of the GRADE assessment is somewhat inconsistent. Although the certainty of evidence is rated as low due to heterogeneity, the Results section simultaneously states that “the majority of studies were of high quality,” which may be confusing. It should be clarified that high methodological quality of individual studies does not compensate for inconsistency and indirectness at the body-of-evidence level.

Response to Reviewer:
We thank the reviewer for this important observation. We have clarified in the manuscript that **although most individual studies were of high methodological quality, the overall certainty of evidence was rated as low due to substantial heterogeneity and indirectness**. This emphasizes that high study-level quality does not fully compensate for inconsistency and indirectness across the body of evidence. And we stated clearly this under the result section. Thank you very much for your insightful comments.
Funding
The Funding section was left empty.

Response to Reviewer:

Dear reviewer thank you for your comment. We noted that funding information was left empty and now we have corrected it.

We sincerely thank the reviewers for their constructive comments, which have helped improve the clarity, rigor, and presentation of the manuscript.
