# Supplementary material for: Global folate deficiency among adolescent girls: A systematic review and meta-analysis
Source: PLoS One. 2026 Apr 20;21(4):e0346599. doi: 10.1371/journal.pone.0346599 (PMC13094969; doi:10.1371/journal.pone.0346599)
Supplement: Attachment — Submitted filename: Resposnse_to_reviewer_auresp_2.docx [file pone.0346599.s007.docx]

**04 March, 2026**

**Dear Editors of PLOS ONE,**

We thank you and the reviewers for the time, effort, and expertise dedicated to the evaluation of our manuscript, PONE-D-25-60905 , entitled “Global Folate Deficiency Among Adolescent Girls: A Systematic Review and Meta-analysis.” We greatly appreciate the constructive and insightful comments provided, which have been invaluable in improving the clarity, rigor, and overall quality of the manuscript.

In response to the reviewers’ feedback, we have carefully revised the manuscript and prepared a detailed, point-by-point response explaining how each comment has been addressed. All modifications have been clearly indicated in the revised manuscript to facilitate review. We believe that these revisions have strengthened the analysis, interpretation, and presentation of our findings.

Thank you for the opportunity to revise and resubmit our work. We appreciate your consideration and look forward to your further assessment.

**Sincerely**

**Mekuriaw Nibret Aweke**

**Corresponding Author**

1. **Editors comment**

Thank you for submitting your manuscript to PLOS ONE. After careful consideration, we feel that it has merit but does not fully meet PLOS ONE’s publication criteria as it currently stands. Therefore, we invite you to submit a revised version of the manuscript that addresses the points raised during the review process.

Authers response:

Thank you for the opportunity to revise our manuscript in response to the editors’ and reviewers’ comments and suggestions. We have revised the manuscript based on the reviewers comment and submitted accordingly. We highly appreciate the editors continues follow up and opportunity to revise the work for better enhancement of the quality of the work..

1. **Revewer #1 comments**

This manuscript addresses an important global public health issue and is generally well structured. The authors have adequately addressed several points raised in the previous round of review, including clarification of methodological procedures, improvements in the presentation of results, and refinement of certain analytical descriptions.

**Response to Reviewer:**

Dear Reviewer, thank you very much for the positive feedback and acknowledgment of the addressing several points raised before. We also highly appreciate the reviewer for the continues reviewing and detail comments and suggesions that improve the quality of the work.

1. Extreme heterogeneity (I² > 99%)

The level of heterogeneity is extremely high. Although subgroup analyses and sensitivity analyses have been performed, the pooled estimate (26.9%) continues to be interpreted in relatively strong terms (e.g., “The findings revealed that 26.9% of adolescent girls are affected by folate deficiency. Approximately one in every four adolescent girls suffers from folate deficiency.”). Given the magnitude of heterogeneity, the manuscript would benefit from a clearer justification for pooling under these conditions and a more cautious interpretation of the overall prevalence estimate. The pooled result should be framed explicitly as a highly heterogeneous summary measure across diverse populations and methodological contexts.

**Response to Reviewer:**

Dear Reviewer,

*Thank you for your suggestion to consider the higher hetrogeniety between studys and interpret the result by considering this higher hetrogeniety.*

*We have revised the above statement and corrected as the following*

*“The pooled prevalence of folate deficiency among adolescent girls was 26.9%. However, this estimate was derived from studies with substantial between-study heterogeneity and should therefore be interpreted as a highly variable summary measure across diverse populations and methodological contexts. On average, approximately one-quarter of adolescent girls were classified as folate deficient, although the true prevalence varied considerably across settings.”*

We hope this revision makes the interpretation of the finding more appropraite and consider the heterogeneity.

2. Biomarkers, Laboratory Methods, and Cut-off Points

The manuscript does not sufficiently explore whether differences in biomarker type (e.g., serum folate versus erythrocyte folate), laboratory methods, or cut-off point definitions were analytically examined as potential sources of heterogeneity. These methodological differences are highly relevant in micronutrient deficiency research and can substantially influence prevalence estimates.

Subgroup analyses or meta-regression incorporating these variables would strengthen the conclusions. If such analyses are not feasible, this limitation should be discussed more explicitly, particularly as a potential contributor to the observed heterogeneity.

**Respon0se to Reviewer:**

Dear Reviewer,

Thank you for suggesting the inclusion of the PECOT framework for our eligibility criteria. We have now added a table presenting the PECOT criteria used in our study. We hope this provides a clear and concise overview of the inclusion and exclusion criteria, and we greatly appreciate your insightful recommendation.

-To assess the degree of agreement between reviewers, it would be useful to calculate Cohen's Kappa index.

**Response to Reviewer:**

Dear Reviewer, we sincerely appreciate your valuable suggestion regarding variations in study cut-off points and laboratory assay methods. Although we conducted a subgroup analysis based on serum type, we were unable to perform subgroup or meta-regression analyses according to cut-off definitions and laboratory assay methods. We have now revised the manuscript to explicitly acknowledge this as a limitation as the following:

“*Another limitation of this review is that variations in laboratory assay methods and cut-off point definitions were not examined through subgroup or meta-regression analyses. These may have contributed substantially to the observed heterogeneity in prevalence estimates*”

3. Integration of Risk of Bias into the Quantitative Analysis
Although a modified version of the Newcastle–Ottawa Scale was applied and adequately described, the analysis does not assess whether study quality influenced the pooled estimate. Incorporating study quality into subgroup analyses or meta-regression, or explicitly discussing the limitations of not integrating quality into the quantitative synthesis, would improve interpretability.

**Response to Reviewer:**

Dear Reviewer, we thank you for your constructive comment regarding the integration of study quality into the quantitative synthesis. Although study quality was assessed using a modified version of the Newcastle–Ottawa Scale, we did not incorporate risk-of-bias scores into subgroup or meta-regression analyses. Due to limited variability in quality ratings and incomplete reporting across some studies, such analyses were not feasible. We have now explicitly acknowledged this as a limitation in the revised manuscript to improve interpretability of the pooled estimate as the following:

“*Study quality was not incorporated into subgroup or meta-regression analyses, which may limit interpretation of the pooled estimate.”*

**Reviewer #2: General Comments**
The authors have adequately addressed the previous comments and revised the manuscript in line with expectations. However, a conceptual issue remains in the PECOT framework. Although the search strategy is appropriate, the PECOT is not fully aligned with it. Specifically, the exposure (E) is not appropriately defined. Folate deficiency represents the outcome of interest rather than an exposure, and listing it as such may be conceptually misleading. In prevalence studies and systematic reviews focused on nutritional status, the exposure typically refers to risk factors associated with the condition, or it may be designated as not applicable (N/A) when the primary objective is purely descriptive. For greater conceptual clarity and methodological consistency, it would be preferable to redefine the exposure as “risk factors for folate deficiency” or to indicate it as N/A.

**Response to Reviewer:**

We sincerely appreciate the positive feedback. We acknowledge that the exposure was not clearly defined, as folate deficiency represents the outcome rather than the exposure. The manuscript has been revised accordingly, and the exposure is now indicated as “Not applicable.”

We are also grateful for the reviewer’s detailed, section-specific comments, which have been invaluable in strengthening the overall quality of the manuscript.
